# Cases of Senile Nerve Degeneration Resembling General Paralysis

**Published:** 1894-12

**Authors:** S. W. MacIlwaine


### CASES OF SENILE NERVE DEGENERATION RESEMBLING GENERAL PARALYSIS.

BY

S. W. MacIlwaine, M.R.C.S. Eng., L.R.C.P. Lond.

The desire to define a disease by giving it a name and sharp boundaries is natural, especially among those who devote their attention to some particular group of diseases. The result in the case of General Paralysis, as in many others, seems to me to be of very doubtful utility.

No doubt it is easy to select cases in an asylum which will form a well-defined group under the name of General Paralysis; but when one has to watch all cases which present themselves in general practice, the boundaries are not at all so easy to maintain. The following cases will serve to illustrate this:—

Case I.—Farmer, æt. 75. First epileptic attack occurred nearly twenty years ago: almost complete intermission of symptoms followed for twelve years; then more and more frequently recurring attacks, with mental and bodily breakdown, ataxia, tremors, unequal pupils, transient albuminuria, delusions, hallucinations, maniacal excitement, with gradual and almost complete extinction of mental and bodily power. In this state he now remains, violent epileptic attacks occurring at intervals of from six weeks to three months.

Case II.—Shopkeeper, æt. 79. Failure of business capacity about fifteen years before death, change in handwriting, loss of memory, prolonged fainting fits; and during the last five years very gradual extinction of all muscular and mental power, wasting, rigidity and contraction of muscles, mental oblivion, retention of urine, cystitis and bedsores.

Case III.—Farmer, æt. 63. History of fainting fit six years ago; first seen eighteen months ago for headache, general weakness and despondency. Tremor of tongue and lips very marked, otherwise nothing amiss discoverable; mind, speech, gait, reflexes, sensation, urine, and optic discs normal, but prognosis clearly bad. Six months later suddenly attacked with aphasia, lasting half-an-hour and followed by hemiparesis; several similar attacks have occurred since, and there is now general muscular weakness with some ataxia and loss of memory. There has been transient double vision.

Case IV.—Shopkeeper, æt. 88. Mental and muscular failure more marked than in normal old age. Had been for many years subject to attacks of prolonged and absolute loss of consciousness, not accompanied by convulsions or followed by hemiplegia. Fifteen months before death there was a violent epileptic seizure, followed by hemiplegia; this quite cleared up in a few weeks, but general mental and bodily power was much impaired. After an interval of eleven months a similar attack occurred; paralysis again cleared up, but left patient on a very low level. After a few weeks an attack of transient aphasia, without convulsions, occurred; and after a similar interval another epileptic attack, followed by coma, Cheyne-Stokes respiration, and death.

Case V.—Farmer, æt. 91. A case of almost normal old age till five years or so before death, when peculiar attacks first occurred, at long intervals, in which there was very marked cyanosis, with a feeling of faintness. Senility advanced more rapidly since then, especially during the last twelve months, and in the last six weeks of life several epileptic attacks occurred; finally, four came on in quick succession, followed by coma, Cheyne-Stokes breathing, and death.

IV. and V. were brothers.

These cases would clearly not come within the pale of general paralysis, according to most writers on the subject; at the same time, I cannot see how general paralysis is to be fairly defined so as to exclude them. Do they not form in fact a series of links or stages between the typical general paralysis of the asylums and normal old age?

There is an observation in Wilks and Moxon's *Pathology* that has not attracted the attention it deserves in this connection. The authors there state that "it is difficult to say in what respect the morbid changes found in the brains of general paralytics differ from those which naturally occur in old age." This means that in reaching a state of ordinary senility there are agencies at work which by slow degrees and without marked symptoms produce, in the course of many years, organic changes in the encephalon indistinguishable from those produced in a couple of years by general paralysis.

The normal oscillations of the cerebral circulation between rest and activity require many years to produce the thickening of membranes, &c., that the terrible congestive waves corresponding to the exaltations of a typical general paralytic can produce in less than three years; but the pathological results are indistinguishable. Are we not justified, then, both on clinical and on pathological grounds, in looking on general paralysis as a premature and rapid senility, rather than as a specific disease? If this is so, may we not hope that it may be possible to recognise a functional stage of general paralysis in which the abnormal congestive attacks may be prevented, and their inevitably fatal organic consequences be forestalled and averted? Indeed, every one can recall cases where it is at least reasonable to suppose that a well-timed holiday may have saved the overwrought city man from general paralysis.

